# Novel Therapeutical Approaches to Managing Atherosclerotic Risk

**DOI:** 10.3390/ijms22094633

**Published:** 2021-04-28

**Authors:** Rosaria Vincenza Giglio, Anca Pantea Stoian, Khalid Al-Rasadi, Maciej Banach, Angelo Maria Patti, Marcello Ciaccio, Ali A. Rizvi, Manfredi Rizzo

**Affiliations:** 1 Department of Biomedicine, Neuroscience, and Advanced Diagnostics, Institute of Clinical Biochemistry, Clinical Molecular Medicine and Laboratory Medicine, University of Palermo, 90127 Palermo, Italy; rosaria.vincenza.giglio@alice.it (R.V.G.); marcello.ciaccio@unipa.it (M.C.); 2 Diabetes, Nutrition and Metabolic Diseases Department, Faculty of General Medicine, Carol Davila University, 050474 Bucharest, Romania; anca.stoian@umfcd.ro; 3 Medical Research Centre, Sultan Qaboos University, Muscat 123, Oman; k.alrasadi@gmail.com; 4 Department of Hypertension, Chair of Nephrology and Hypertension, Medical University of Lodz, 90-419 Lodz, Poland; maciej.banach@umed.lodz.pl; 5 Polish Mother’s Memorial Hospital Research Institute, 93-338 Lodz, Poland; 6 Cardiovascular Research Centre, University of Zielona Gora, 65-417 Zielona Gora, Poland; 7 Department of Health Promotion, Mother and Child Care, Internal Medicine and Medical Specialties, University of Palermo, 90133 Palermo, Italy; manfredi.rizzo@unipa.it; 8 Department of Laboratory Medicine, University-Hospital, 90127 Palermo, Italy; 9 Division of Endocrinology, Metabolism, and Lipids, Department of Medicine, Emory University, Atlanta, GA 30322, USA; ali.abbas.rizvi@emory.edu; 10 Division of Endocrinology, Diabetes and Metabolism, School of Medicine, University of South Carolina, Columbia, SC 29208, USA

**Keywords:** atherosclerosis, inflammations, oxidative stress, innovative therapies, nutraceuticals, molecular signaling, management

## Abstract

Atherosclerosis is a multifactorial vascular disease that leads to inflammation and stiffening of the arteries and decreases their elasticity due to the accumulation of calcium, small dense Low Density Lipoproteins (sdLDL), inflammatory cells, and fibrotic material. A review of studies pertaining to cardiometabolic risk factors, lipids alterations, hypolipidemic agents, nutraceuticals, hypoglycaemic drugs, atherosclerosis, endothelial dysfunction, and inflammation was performed. There are several therapeutic strategies including Proprotein Convertase Subtilisin/Kexin 9 (PCSK9) inhibitors, inclisiran, bempedoic acid, Glucagon-Like Peptide-1 Receptor agonists (GLP-1 RAs), and nutraceuticals that promise improvement in the atheromatous plaque from a molecular point of view, because have actions on the exposure of the LDL-Receptor (LDL-R), on endothelial dysfunction, activation of macrophages, on lipid oxidation, formations on foam cells, and deposition extracellular lipids. Atheroma plaque reduction both as a result of LDL-Cholesterol (LDL-C) intensive lowering and reducing inflammation and other residual risk factors is an integral part of the management of atherosclerotic disease, and the use of valid therapeutic alternatives appear to be appealing avenues to solving the problem.

## 1. Introduction

Atherogenesis at the arterial wall level is an accumulation of intracellular lipids accompanied by stimulation of proliferation and increased synthesis and secretion of extracellular matrix and induction of the synthesis and secretion of inflammatory cytokines [1].

These changes lead to the formation of foci of chronic inflammation and morphologically unstable plaques that are prone to rupture, causing clinical manifestations of atherosclerosis [2].

Low Density Lipoproteins (LDL) are atherogenic components in the bloodstream, present in large quantities in atherosclerosis patients. The discovery of the subclasses of small and dense LDL (sdLDL) in recent years has determined the so-called phenomenon of serum atherogenicity, i.e., the ability to induce lipids accumulation in the cells of the arterial intima [3,4,5]. Oxidized LDLs are independent predictors of subclinical and clinical atherosclerosis; oxidative modifications of sdLDL represent an early stage of atherosclerosis and sdLDL are more susceptible to oxidation than larger and wider particles [6].

Local inflammation is considered one of the possible contributing causes in the development of atherosclerotic lesions in the arterial wall, while inflammatory cytokines at all stages of atherosclerotic lesion formation play significant roles in the clinical manifestations of atherosclerosis [7].

Timely prevention is imperative in people with subclinical atherosclerosis to retard the development of serious complications such as stroke, acute coronary syndrome, and other life-threatening atherosclerotic diseases. Early detection is carried out by ultrasound screening of the central and peripheral arteries and subsequently, especially in the heart, by computed tomography to evaluate coronary arteries and intracoronary calcium content [6,7]. Since atherosclerosis develops over many years, it is necessary to take into account the need for long-term anti-atherosclerotic therapy, taking into consideration the use of drugs or nutraceuticals that act on the various molecular targets of atherosclerosis [8,9], without causing side effects that might lead to their suspension [10].

The pathogenetic approach to the prevention of arteriosclerosis in its early stages includes the suppression of cholesterol accumulation in the arterial wall cells almost in a tailor-made manner, based on the molecular cluster that must be silenced or reduced. Considering stable and unstable plaque, there is a different therapeutic treatment based on the percentage of plaque (within 70% of drug treatment, over 70% surgical and drug approach).

This narrative review aims to evaluate the new therapeutic options that act directly or indirectly on atherosclerosis from a molecular point of view.

## 2. Methods

We performed an electronic database search (MEDLINE (1975–November 2020), EMBASE and SCOPUS (2000–November 2020)) assessing the association between novel nutraceuticals, hypolipidemic agents, and hypoglycemic drugs and molecular effects on atherogenesis.

## 3. Results

### 3.1. Innovative Therapies with Direct Action on Atherosclerosis

The infiltration of LDL particles into the arterial wall is the trigger event leading to the development of atherosclerosis [11]. Treatments aimed at reducing Low Density Lipoproteins-Cholesterol (LDL-C) are statins, inhibitors of the 3-Hydroxy-3-MethylGlutaryl Coenzyme A (HMG CoA) reductase, which exert their primary therapeutic effect by upregulating the LDL-Receptor (LDL-R). Statins are generally well-tolerated, safe, and affordable. Suppose despite statin therapy, the patient does not get to target or manifests side effects from statin intolerance (myalgia/myopathy, rhabdomyolysis, temporary elevation of alanine aminotransferase, new onset diabetes) [10,12]. In that case, the use of more aggressive therapies is recommended for patients with particularly high-risk primary and secondary prevention [13]. Taking into account the new data with the above and below mentioned therapies, for high to extremely high-risk patients we focus not only to the lower the better approach, but also the earlier the better and the longer the better (preferably lifelong on LDL-C target) [14,15,16]. Completely another issue is related to residual risk, and all described below innovative therapies might help in order to effectively fight both LDL and residual-related CardioVascular Disease (CVD) risks.

### 3.2. Proprotein Convertase Subtilisin/Kexin Type 9

Proprotein Convertase Subtilisin/Kexin 9 (PCSK9) monoclonal antibodies are a breakthrough for statin-intolerant patients or unable to achieve LDL-C goals with statins [17]. PCSK9 inhibitors provide a useful option for managing LDL-C and residual CardioVascular (CV) risk in high-risk patients: PCSK9 inhibitors reduce LDL-C by 50–70%. PCSK9 is known as an enzyme that binds to the hepatocyte LDL-R, leading to its degradation and increased expression of the LDL-R on hepatocytes, thus promoting clearance of LDL-C by the liver [18]. In the latest American College of Cardiology/American Heart Association (ACC/AHA) guidelines, PCSK9 inhibitors are recommended in high-risk secondary prevention patients who are on maximally tolerated statin therapy but have LDL-C greater than 70 mg/dL, and in patients with severe primary hypercholesterolemia (LDL-C > 190 mg/dL) or heterozygous Familial Hypercholesterolemia (FH) who are not at the goal despite maximally tolerated therapy [19].

PCSK9 inhibitors are used for high-risk patients (for secondary prevention or severe primary hypercholesterolemia) who have statin intolerance syndrome or who have an insufficient reduction in LDL-C level while taking the maximum tolerated dose of a statin plus ezetimibe [8]. There appears to be a linear relationship between reduced LDL-C level and lower CV risk, even down to LDL-C values below 10 mg per deciliter (0.3 mmol per L), with no early signal of harm associated with levels of very low LDL-C [20].

PCSK9 inhibitors have been tested for CV risk relief in two major trials: the “Further Cardiovascular Outcomes Research with PCSK9 Inhibition in Subjects with Elevated Risk (FOURIER)” study of evolocumab and the “Safety and Tolerability of Alirocumab in High Cardiovascular Risk Patients with Hypercholesterolemia Not Adequately Controlled with Their Lipid Modifying Therapy (ODYSSEY)” study of alirocumab. In a pooled analysis of four phase 3 studies, the efficacy and safety of evolocumab were comparable in patients with or without Type 2 diabetes mellitus (T2DM) and did not differ between T2DM subgroups [21]. The FOURIER study [22] enrolled approximately 27,000 subjects with stable atherosclerotic CVD and an LDL-C level of 70 mg per deciliter or higher, or a cholesterol level not High Density Lipoprotein (HDL) of 100 mg per deciliter (2.6 mmol per L) or higher treated with high or moderate-intensity statin therapy. In this study, the primary outcome was the reduction in Major Adverse Cardiovascular Events-MACE (death from CV causes, myocardial infarction, stroke, hospitalization for unstable angina, or coronary revascularization); there was a reduction in mean LDL-C level of 30 mg per deciliter (0.8 mmol per L) and a reduction in MACE in people treated with evolocumab especially in patients with a higher absolute risk of these events, such as people with peripheral artery disease, a history of recent or multiple myocardial infarctions, or elevated levels of Lipoprotein a (Lp(a)) [22].

In the “Open-Label Study of Long-Term Evaluation Against LDL Cholesterol (OSLER)” I and the OSLER II studies, in high and very high CV risk subjects on statin therapy with or without ezetimibe or ezetimibe alone, evolocumab 140 mg every two weeks or 420 mg monthly as a subcutaneous injection for 11.1 months showed a significant reduction in LDL-C (−61%, *p* < 0.001) compared to standard therapy alone at week 12 [23].

The ODYSSEY OUTCOMES study tested the benefit of alirocumab in patients with previous acute coronary syndrome within the past year and with LDL-C values of at least 70 mg per deciliter, a non-HDL cholesterol level of at least 100 mg per deciliter, or a level of Apolipoprotein B (Apo B) of at least 80 mg per deciliter treated at the maximum tolerated dose of a statin [24]. The study involved a dose adjustment to achieve a reduction in LDL-C of 25–50 mg per deciliter (0.6 to 1.3 mmol per L). In fact, a decrease in LDL-C of 38 mg per deciliter (1.0 mmol per L) was found post-treatment to be associated with a significant absolute reduction in risk of 15% for MACE.

In the “Long-term Safety and Tolerability of Alirocumab in High Cardiovascular Risk Patients with Hypercholesterolemia Not Adequately Controlled with Their Lipid Modifying Therapy (ODYSSEY LONG-TERM)”, the effects of alirocumab were coherent with previous studies of alirocumab with a significant reduction in LDL-C in the group treated with alirocumab 150 mg subcutaneously every two weeks for 78 weeks versus the placebo group after 24 weeks at the maximum tolerated dose of statins with or without ezetimibe [25].

In the study of efficacy and safety of alirocumab vs. ezetimibe in statin-intolerant patients with a statin rechallenge arm (ODYSSEY ALTERNATIVE), alirocumab showed a significant reduction in LDL compared to ezetimibe in subjects with primary hypercholesterolemia and moderate, high, or very high CV risk, who were intolerant to statins (*p* < 0.0001) [26].

Inclisiran (small interfering RNA molecule) acts with a completely new mechanism of inhibition of the PSCK9 protein in hepatocytes, targeting the messenger RNA for PCSK9 [27].

In “Sustained LDL-C Reduction with Inclisiran (ORION-1)” [28], a phase 2 study, 501 high-risk CVD patients with elevated LDL-C levels, despite the maximum tolerated dose of statins, were randomized to take one dose of placebo or 200, 300, or 500 mg of inclisiran (a long-acting PCRK RNA PCSK9 messenger RNA that specifically cleaves the coding of PCSK9 mRNA) [29] administered subcutaneously as one or two doses (on days 1 and 90) of placebo. Inclisiran reduced PCSK9 and LDL-C levels in a dose-dependent manner (from 27.9 to 41.9% after a single dose and from 35.5 to 52.6% after two doses (*p* < 0.001 for all comparisons vs. placebo)) [28]. Inclisiran has just been approved by the European Medicines Agency (and previously by the FDA) and in 2021 will be available in EU countries.

PCSK9 inhibitors are currently recommended in the following patient categories: very high-risk secondary prevention patients who are on maximally tolerated statin therapy but have LDL-C greater than 70 mg/dL; patients with statin intolerance and patients with severe primary hypercholesterolaemia (LDL-C > 190 mg/dL), or heterozygous FH who fail despite maximally tolerated therapy [8]. In clinical practice, PCSK9 inhibitors might also be used in selected “primary prevention” patients with multiple risk factors, with statin intolerance syndrome and in patients with evidence of significant coronary atherosclerosis. Unfortunately, due to reimbursement statements (mostly within drug programs) their usage is limited in most of the countries; besides, oral PCSK9 inhibitors are not available so they have to be injected subcutaneously, which makes them much less practical than statins that are easily administered orally [30].

### 3.3. Bempedoic Acid

Bempedoic acid is an Adenosine TriPhosphate (ATP) citrate lyase inhibitor that upregulates LDL-R by reducing cholesterol synthesis [31]. It is important to emphasize that it is prodrug in muscles, and it is in this active form in hepatocytes. There is a direct correlation between LDL-C and the processes of plaque formation. The oxidation of LDL-C and subsequent incorporation into the walls of the arteries trigger inflammation of the arterial walls, the weakening of the atherosclerotic plaque and subsequent acute cardiovascular event; one of the main indices of inflammation is high sensitivity-C Reactive Protein (hs-CRP) [32]. The pivotal studies of bempedoic acid showed a reduction not only in LDL-C but also in hs-CRP [31,32,33,34]. That is why it might be a very useful and effective tool in statin intolerant patients. In the CLEAR Harmony (Evaluation of Long-Term Safety and Tolerability of ETC-1002 in High-Risk Patients with Hyperlipidemia and High Cardiovascular Risk) study, patients who received maximally tolerated statin therapy administered with bempedoic acid had LDL-C levels significantly lower than placebo (mean difference, 18%), without an increase in serious adverse events [32]. Based on the pooled analysis of phase 3 trials in might reduce LDL-C by even 25% (is much more effective in statin intolerant patients) CRP by even 40% with acceptable safety profile [31,32,33,34].

Cardiovascular outcomes study is still ongoing; CLEAR-OUTCOMES (Evaluation of Major Cardiovascular Events in Patients with, or at High Risk for, Cardiovascular Disease Who Are Statin Intolerant Treated With Bempedoic Acid (ETC-1002) or Placebo) is enrolling high-risk CV patients who have adverse effects in response to statins and have an LDL-C level of 100 mg per deciliter or higher [33].

### 3.4. Nutraceuticals

The nutraceutical combination together with a cholesterol-lowering action, associated with an adequate lifestyle, provides an alternative to pharmacotherapy in patients who report intolerance to statins and in subjects with low cardiovascular risk [9].

Several nutraceuticals positively modulate lipid metabolism [35]: plant sterols and soluble fibers decrease intestinal assimilation of lipids and increase their elimination and reduce sdLDL [36]; berberine and soy proteins improve the absorption of cholesterol in the liver and act by improving endothelial dysfunction [37]; policosanols, monacolins, and bergamot inhibit the enzymatic action of HMGCoA reductase and reducing atherogenic sdLDL [38].

Coenzyme Q10 (CoQ10) is a vitamin-like compound widely distributed in the body [39]. CoQ10 performs several functions but is important as it acts as a coenzyme in mitochondrial energy production, stabilizing the membrane and preventing lipid peroxidation [40]. In statin intolerance syndrome, mitochondrial dysfunction could be induced by a CoQ10 deficiency [41]. The use of this enzyme in patients with statin intolerance syndrome is useful for restoring muscle function and assists low-dose statins in reducing cholesterol [10].

Despite the available pharmacotherapies, the onset of atherosclerosis is determined by multiple cell signaling pathways. The crucial role is probably due to the Notch receptors that regulate the functions of the different types of cells involved in the onset progression of atherosclerosis [42]. Notch signaling is a short intercellular communication system studied in neoplasms as a therapeutic target, but it is becoming a new key to the maintenance of vascular homeostasis in atherosclerosis; according to the “traction force” theory, Notch signaling is activated when the MindBomb E3 ubiquitin protein ligase 1 (MIB1) modifies the Notch ligands, allowing the ligand to endocytosis and generating the mechanical force needed to expose the second Notch receptor cleavage site [43]. Several structural changes and interaction with Nuclear Factor kappa-light-chain-enhancer of activated B cells (NF-κB), Estrogen Receptor- (ER-) alpha, ERG Protein-coupled (GPER), Human Epidermal growth factor Receptor 2 (HER-2 also known as ErbB2) and Vascular Endothelial Growth Factor Receptors (VEGFR) increase complexity of these signaling pathways that influence atherosclerosis [43]. Notch has been in the spotlight because Notch can counteract endothelial dysfunction and the development of atherosclerotic plaque. Tumor Necrosis Factor (TNF)-α disrupts Notch signaling, leading to increased levels of InterCellular Adhesion Molecule 1 (ICAM-1) and Vascular Cell Adhesion Molecule protein 1 (VCAM-1) and NF-κB-mediated apoptosis [44]; siRNA-mediated reduction of Notch-1 is enough to increase the expression of inflammatory markers and adhesion molecules; oxidized lipids and TNF-α and InterLeukin 1 beta (IL1β) decreased Notch-1 expression [45]. Several Notch signaling components in the endothelium respond to shear stress [46]; Notch-1 can be activated by shear stress through a mechanism dependent on Delta-like 4, a protein that codes for Notch ligands, triggering a noncanonical Notch path [47].

Different classes of nutraceuticals could modulate Notch’s pathways. Olive oil improved plasma HDL levels, decreased the level of systemic EndoThelin-1 (ET-1), enhanced HDL content, increased endogenous antioxidant enzymes, reduced DNA oxidation level, increased fecal microbial metabolic activity, ameliorated endothelial function [48]; for flavonoids, epicatechin improved endothelial function and reduced inflammation [49] and EpiGalloCatechin-3-Gallate (EGCG) restores the expression of Jagged-1 (the key effector of EGCG-protective effect against oxLDL-induced endothelial dysfunction) and of target proteins (MATH1, HES1, and HES5) [50]; norisoboldine induces VEGF-mediated migration through activation of Notch-1 [51]; DocosaHexaenoic Acid (DHA) increases Notch-3 expression and HES1 transcription and enhances γ-secretase complex activity [52]; Diosgenin prevents nuclear translocation of NICD in aorta and in differentiated macrophage cells [53]; BerBeRine (BBR) upregulates NICD translocation and HES1 expression and in vitro antiapoptotic effect of BBR is blocked by Notch-1 or HES1 siRNA [54]; polydatin exerts cardioprotection against diabetic myocardial IRI by activating myocardial Notch-1/HES1 signaling, DAPT blunts the beneficial effects of polydatin [55]; 2,3,5,4′-TetrahydroxyStilbene-2-o-β-d-Glucoside (TSG) upregulates NICD and HES1 expression and in vitro, antiapoptotic effect of TSG is blocked by gamma-secretase DAPT [56]; resveratrol decreases Notch-1, Jagged-1, Hey1, and Hey2 mRNA in balloon-injured arteries at 7 days [57].

Based on above, we might confirm that nutraceuticals, especially those with confirmed properties and safety (nutrivigilance), might have many different beneficial properties, working a bit similarly to statins (=pleiotropic effects), not only related to LDL-C reduction, but also glucose, weight, and blood pressure reduction, improvement of the endothelial function and arterial stiffness with the reduction of inflammation and oxidative stress. However, we need to remember that they might be added as an add-on to therapy and cannot replace this [58,59,60].

### 3.5. Glucagon-Like Peptide-1 Receptor Agonists

Glucagon-Like Peptide-1 Receptor agonists (GLP-1 RAs) may favorably affect CV risk through direct actions on the myocardium and blood vessels [61], and it has been shown that liraglutide may exert significant effects at the early stage of atherosclerosis and slows its progression [62]. GLP-1 RAs are used in diabetic patients but liraglutide is also approved for the treatment of obesity; interestingly, liraglutide has been shown to improve lipid and lipoprotein profile, and sdLDL, in a rigorous randomized, placebo-controlled, cross-over, double-blind study on patients with obesity, regardless the presence of diabetes [63]. Circulating LDL molecules are transported from the vascular space into the arterial wall and retained in the extracellular matrix, where they are prone to form oxidized LDL that contributes to atherosclerotic plaque formation [62]; therefore, the very early stages of atherosclerosis include the increased arterial entry and retention of LDL because of their decreased clearance by LDL-R in plasma, with increased oxidation of LDL in the arteries and greater endothelial dysfunction, foam cell formation, and the appearance of carotid intima-media thickness as early carotid lesions [62]. Interestingly, it has been shown that in patients with early stages of atherosclerosis, endothelial dysfunction and carotid lesions are strong predictors of future clinical events [64].

Liraglutide decreased atherosclerotic lesion formation in ApoE−/− mice coincident with a reduction in pro-inflammatory and increased anti-inflammatory monocyte/macrophage populations in vivo; analysis of macrophages for MΦ1 (pro-atherogenic) and MΦ2 (pro-resolving) macrophage markers, showed that liraglutide modulates macrophage cell fate towards MΦ2 pro-resolving macrophages [65]. Liraglutide and semaglutide significantly attenuated plaque lesion development in Apolipoprotein E (ApoE)−/− and LDL-R−/−) deficient mice; semaglutide treatment improved leukocyte recruitment, leukocyte rolling, adhesion/extravasation, cholesterol metabolism, lipid-mediated signaling, extracellular matrix protein turnover, and plaque hemorrhage [66]. Liraglutide improves metabolic parameters and carotid intima-media thickness in diabetic patients with the metabolic syndrome [67] and, very recently, it has been shown a novel anti-atherogenic effect of liraglutide in patients with type-2 diabetes: the decrease in subclinical atherosclerosis by a reduction in atherogenic small, dense LDL particles [68]. Semaglutide treatment significantly lowered fasting and postprandial lipid metabolism vs. placebo [69].

### 3.6. Interleukins: A Potential Therapeutical Target

Inflammation mediates all stages of atherosclerotic disease and it is involved in the progression of atherosclerotic lesions [2]. It has been shown that the characteristics of plaque instability are related to the upregulation of several pro-inflammatory markers such as cytokine interleukin-6 (IL-6) and TNF-α, endothelial activation markers, such as hs-CRP, long pentraxin-3 [70], the IL-1 receptor family [71], serum levels of circulating Matrix MetalloProteinase (MMP), Tissue Inhibitor of MetalloProteinase-1 (TIMP-1), and IL-8 [72].

The shortening of leukocyte telomeres, an indicator of leukocyte activity and replicative capacity, has been associated with a greater progression of the intima-media thickness indicating subclinical vascular damage [73]. In patients with atherosclerosis, mononuclear cells, lymphocytes, and monocyte subpopulation are associated with plaque progression and vulnerability [74]: association between the presence of plaque and total white blood cells and monocyte counts [75], neutrophil count correlates with the presence of microembolism, increased activation of T and B lymphocytes, and higher levels of MMP-9 expression in peripheral blood mononuclear cells [76].

Few studies have focused on the effect of anti-inflammatory drugs alone on cardiovascular risk, but some drugs used in the prevention of atherosclerosis, such as antiplatelet agents and statins, also have anti-inflammatory effects [77]. Aspirin and clopidogrel act on inflammation, and the latter when used at high doses [78]; aspirin inhibits cyclooxygenase and pro-inflammatory signaling pathways, including NF-κB [79]. In the “Justification for Use of Statins in Prevention: A Rosuvastatin Assessment Intervention Test” (JUPITER) study, it was shown that statins are able to reduce circulating and intraplaque inflammation in normolipidemic subjects with high levels of inflammatory markers, regardless of cholesterol values [80].

Recently, new anti-inflammatory treatment strategies of atherosclerosis have been introduced. Some of these drugs have recently been used for the suppression of the inflammatory cascade induced by some viruses, others for the treatment of inflammatory bowel diseases, and some others for systemic autoimmune inflammatory diseases [81,82]. Tocilizumab, a monoclonal antibody that blocks IL-6 receptors, has reduced myocardial damage and systemic inflammation, although there was a serious safety concern due to a significant increase in LDL-cholesterol levels soon after starting treatment [83]. Furthermore, canakinumab, a human monoclonal antibody targeted against IL-1β that has anti-inflammatory effects, in the “Canakinumab Anti-Inflammatory Thrombosis Outcomes Study” (CANTOS), reduced recurrent cardiovascular events compared to placebo in patients with a history of myocardial infarction and a high degree of hs-CRP, regardless of the lipid lowering effect [84]. Further, colchicine inhibits caspase-1 proteolysis and IL-1β secretion in macrophages, and at low doses reduced the risk of major cardiovascular events [85]. Finally, the TNF-α neutralizing antibody, infliximab, improved endothelial function and reduced adhesion molecules [86].

Nanoimmunotherapy moderates CD40-CD40 ligand signaling in monocytes and macrophages by blocking the interaction between CD40 and Tumor necrosis factor Receptor Associated Factor six (TRAF6). In apolipoprotein E-deficient (Apoe−/−) mice, a 1 week nanoimmunotherapy treatment regimen achieved significant anti-inflammatory effects, due to the reduced migration capacity of plaque monocytes; this highlights the translational potential of this strategy for the treatment of atherosclerosis [87].

### 3.7. Atherogenic Lipoproteins: A Possible Therapeutic Target

In patients who develop an unexpected cardiovascular event, it is important to perform a thorough diagnostic evaluation, including the study of atherogenic lipoproteins. In this context, Lp(a), has been proposed over the years as an independent risk factor for the development and progression of atherosclerosis and cardiovascular disease, although data are not fully consistent [88]. Similar in structure to that of LDL-C particles, differentiated by the presence of Apolipoprotein A (ApoA) covalently linked to apolipoprotein B (apoB), high levels of Lp(a) exert a potentially more pronounced pro-atherogenic action on the vascular endothelium than that exerted by LDL-C, due to the high binding affinity of the lipoprotein to the vascular endothelium and the pro-inflammatory and pro-thrombotic effects related to apo(a) [89]. Lp(a) may represent a possible therapeutic target in patients with a rapid progression of atherosclerotic disease. The marked increase in the risk of events observed in patients with very high Lp(a) levels (above 50 mg/dL) suggests possible therapeutic implications for the reduction of Lp(a) levels [90]. Traditional oral lipid-lowering therapies cause a reduction of no more than 20% in circulating Lp(a) levels [91]; PCSK9 inhibitors demonstrated a modest effect in reducing Lp(a) [25]. The new AntiSense Oligonucleotides (ASO) anti-apo(a) block the assembly of Lp(a) and reduce its plasma levels by over 70% [92], while lipoprotein apheresis is able to reduce Lp(a) levels by more than 50% [93].

LDL are considered main risk factors for CVD, while HDL are known for their atheroprotective role, but ethnic differences in serum lipoproteins and their determinants exist [94]. Both LDL and HDL are heterogeneous in nature, including various subfractions depending on the isolation method, with up to 7 and up to 10 distinct different subspecies for LDL and HDL, respectively [4]. The predominance of sdLDL has been shown over the years to be an important and independent predictor of cardiovascular events and progression of coronary heart disease, even independently of LDL-cholesterol concentrations [3,4,95]. Therefore, it has become evident that the quality rather than only the quantity of LDL is closely related to cardiovascular risk, and estimating sdLDL in clinical practice helps to identify patients at higher risk of future cardiovascular events and helps to direct specific preventive measures [11]. While sdLDLs have fully elucidated atherogenic potential, the role of the different HDL subfractions is still largely unclear, as the atheroprotective role of HDL particles differs according to their size [3].

Several studies have shown that therapeutic modulation of LDL size is of great benefit in reducing the risk of cardiovascular events and that emerging therapies for raising HDL cholesterol and augmenting HDL particle functionality are needed [96]. Small, dense LDL represent a main feature of the Metabolic Syndrome (MetS), their levels are abundant in patients with different MetS profiles, even independent of the presence of type 2 diabetes [97,98,99], and their predictive has been clearly assessed; sdLDL are strong and independent predictors of cardiovascular and cerebrovascular events in subjects with the metabolic syndrome [100]. Lipid-lowering drugs, and antidiabetic agents and nutraceuticals, exert favorable effects on these atherogenic particles [3] and there is continuing interest in the development of new therapeutic approaches aiming to reduce the atherogenic potential of sdLDL particles [101]. This would probably reduce large excess cardiovascular risk found in clinical practice in many patients.

### 3.8. Glucose-Dependent Insulinotropic Polypeptide (GIP)

Glucose-dependent Insulinotropic Polypeptide (GIP) is the intestinal hormone secreted by enteroendocrine K cells in response to digested nutrients [102]. It is considered an incretin due to its ability to stimulate insulin secretion from pancreatic beta cells in a glucose-dependent manner [103]. The role of GIP in cardiovascular disease is yet to be clarified; pharmacological doses of GIP-Receptor agonists (GIP-RA) have been found to exert antiobesity effects in animal models [104]. Studies on different cell lines and animals, and very few studies conducted on patients, have shown a possible protective action in the onset and evolution of atheromatous plaque.

The GIPR signaling pathway in Vascular Endothelial Cells (VEC) is perhaps distinct from the pancreatic beta cell signaling pathway, while the effects of GIP may differ between vascular or inflammatory cell types. In VECs, GIPR increases Nitric Oxide (NO) production [105], reduces Advanced Glycation End-product (AGE)-induced oxidative stress and inflammation [106], increases NO production [107], increases Adenosine Monophosphate Protein Kinase (AMPK) activation [108], and reduces Inducible Nitric Oxide Synthetase (iNOS) level [107]; in Vascular Smooth Muscle Cells (VSMC) it reduces cell proliferation induced by growth factor [109]; at the monocyto–macrophage level it reduces inflammation [110] and their migration [111]; at the adipocyte level it increases the levels of adiponectin [112] and modulates inflammation [113].

In studies conducted in animal models and in the context of atherosclerosis, the activation of GIP has favored the reduction of plaque formation and macrophage foam cells [114], the increase in plaque stability [111], the reduction of formation plaque and foam cell formation of macrophages [109]. In the area of inflammation, it modulated inflammation of the adipose tissue [112], increased expression of adipose tissue and blood levels of adiponectin [105], and reduced blood IL-6 level [115].

In studies conducted on nondiabetic subjects, GIP reduces mean arterial pressure and heart rate [116]; increases femoral artery blood flow [117]; increases blood levels of chemokine C-C motif ligand (CCL)-2 [118], CCL8, and osteopontin [119]; and increases the levels in adipose tissue of CCL2, CCL8, and IL-6 [118]. In studies conducted on patients with dysglycemia or diabetes, it reduces mean arterial pressure and increases heart rate [116].

## 4. Discussion

The link between inflammation and plaque healing remains incompletely understood [120]. The resolution of inflammation in atherosclerosis is mediated by specialized, pro-resolving mediators, such as resolvin, lipoxins, maresine, and protectins. Exposure to interferon-γ inhibits smooth muscle cell ability to produce interstitial collagen that repairs the fibrous cap and maintains integrity [121]. Macrophages are not terminally differentiated in atherosclerotic disease but can switch from one phenotype to another in response to environmental cues [122]. Cross-talk between Th1 cells and macrophages increases interstitial collagenase production, including matrix metalloproteinases 1, 8, and 13, promoting the breakdown of interstitial collagen and weakening the fibrous cap [123]. M2 macrophages classified into three subtypes, M2a, M2b, and M2c, are activated by interleukin-4 and interleukin-13, and secrete interleukin-10, which counterbalances the pro-inflammatory activity of M1 macrophages and promotes tissue repair [122]. The wound-healing M2a macrophages express high levels of scavenger receptors, mannose, and galactose, and produce pro-fibrotic factors, such as fibronectin and insulin-such as Insulin like Growth Factor 1 and TGF-β, which can contribute to plaque healing [124]. In the latter stages of the cure process, M2 macrophages can promote plaque calcification by stimulating osteoblastic differentiation and maturation of vascular smooth muscle cells [125]. Calcium formation during plaque healing is the result of dysregulated deposition and reduced clearance.

**Figure 1:**
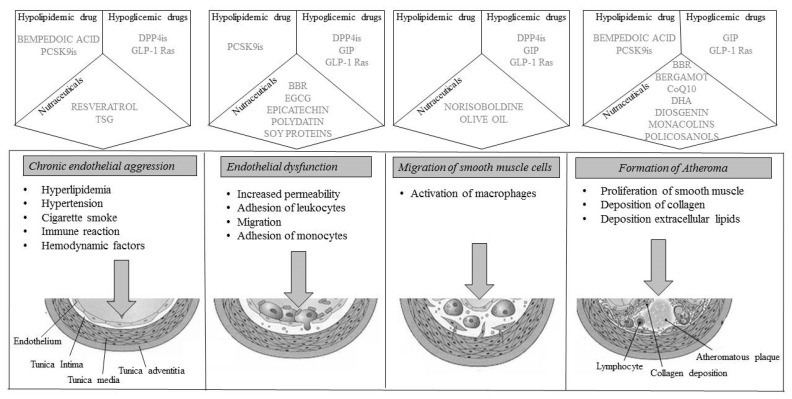
Representation of plaque progression with drug molecular actions. BBR: BerBeRine; CoQ10: Coenzyme Q110; DHA: DocosaHexaenoic Acid; DPP4is: DiPeptidyl Peptidase 4 inhibitors; EGCG: EpiGalloCatechin-3-Gallate; GIP: Glucose-dependent Insulinotropic Polypeptide; GLP-1 Ras; Glucagon Like Peptide-1 Receptor agonists; PCSK9is: Proprotein Convertase Subtilisin/Kexin 9 inhibitors; TSG: 2,3,4,5′-TetrahydroxyStilbene-2-o-β-d-Glucoside.

However, several therapeutic strategies can reliably show an improvement in the atheromatous plaque (Figure 1). First, the lipid-lowering diet and nutraceuticals reduce the expression and proteolytic activity of interstitial collagenase (MMP-1), reduce oxidative stress, and activate endothelial cells and the thrombotic potential. Among the drugs, statins are associated with beneficial modifications in emerging cardiovascular risk factors [126,127,128]; they have a regulatory action on the secretion of adipokines, which can be induced by LipopolySaccharides (LPS) or the general inflammatory state associated with coronary atherosclerotic disease, causing an increased secretion of adiponectin or reducing the levels of the inflammatory cytokine IL-6 [129], reducing leptin levels [130], or reducing Plasminogen Activator Inhibitor -1 (PAI-1) synthesis in adipocytes, also improves coagulation homeostasis [131]; PCSK9 inhibitors, and anti-inflammatories have the same effects and also upregulate circulating endothelial progenitor cells and angiogenic cells.

Pioglitazone inhibits cardiac remodeling induced by angiotensin 2 through adiponectin, of which it also increases the levels [132]; increases larger LDL concentrations both in fasting and after meals, and reduces the levels of small-dense atherogenic particles after meals [133,134,135]; reduces the serum levels of adipokine chemerin [136]; and also the expression of resistin in adipocytes [137]. Among the new hypoglycemic therapies, incretins and gliflozines seem to show a remarkable impact in the modulation of inflammation, reducing endothelial stress and the formation of atheromatous plaque.

In the future, epigenetic therapies and inhibitors of the PI3Kc-CXCL10 axis will likely target macrophages’ polarization towards an alternative M2 phenotype and thus increase re-endothelization after vascular damage.

## 5. Conclusions

Atheroma plaque reduction and stabilization is an integral part of the amelioration of atherosclerotic disease and the use of valid therapeutic alternatives to manage these patients appears to be an adequate alternative to solving the problem (Table 1). Today, the sewn therapy, like a dress on the patient’s needs, is understood as “tailoring therapy”. In contrast, therapies that are considered sectorial (hypoglycemic, lipid-lowering, anti-inflammatory, epigenetic therapies) can be used to augment its therapeutic effects. The patient who is confronted with multiple metabolic risk factors stands to benefit from exploring new therapeutic frontiers to achieve the goal of personalized disease management.

## Figures and Tables

**Table 1:** Illustrative table with novel therapeutic approaches useful managing atherosclerotic risk.

Pharmacological Class	Drug	Effect on Atherosclerotic Risk	References
	Statins	↓ Infiltration of LDL particles into the arterial wall	[12,13,14,15,16]
Lipid-lowering agents	PCSK9	↑ Expression of the LDL-R on hepatocytes promoting clearance of LDL-C by the liver; Stabilization and plaque regression	[17,18,19,20,21,22,23,24,25,26,27,28,29,30]
	Bempedoic acid	↓ Cholesterol synthesis; ↓ Indices of inflammation	[31,32,33]
Hypoglicemic agents	GLP-1 RAs	Direct actions on the myocardium and blood vessels; Significant effects on the early stage of atherosclerosis, arterial entry and retention of LDL; ↓ Subclinical atherosclerosis by a reduction in atherogenic small, dense LDL particles	[62,63,65,66,67,68]
	GIP	Protective action in the onset and evolution of atheromatous plaque; ↓ Plaque formation and macrophage foam cells; ↑Plaque stability; ↓ Mean arterial pressure	[101,102,103,104,105,106,107,108,109,110,111,112,113,114,115]
	Tocilizumab	Blocks IL-6 receptors; ↓ Myocardial damage and systemic inflammation	[82]
Anti-inflammatory agents	Colchicine	Inhibits caspase-1 proteolysis and IL-1β secretion in macrophages	[84]
	Canakinumab	Improved endothelial function; ↓ Adhesion molecules	[83]
	CoQ10	Preventing lipid peroxidation	[39,40,41,42]
	Soy proteins	Improving endothelial dysfunction	[37,38]
	Olive oil	↓ Level of systemic ET-1; ↑ Endogenous antioxidant enzymes; ↓ DNA oxidation level; Ameliorated endothelial function	[48]
	Epicatechin	Improved endothelial function and reduced inflammation	[49]
	EGCG	Restores the expression of Jagged-1, the key effector of EGCG-protective effect against oxLDL-induced endothelial dysfunction	[50]
Nutraceuticals	Norisoboldine	Effect on inflammasoma	[51]
	DHA	Effect on vessel wall shear stress and atherosclerosis	[52]
	Diosgenin	Preventing differentiated macrophage cells; ↓ Level of oxLDL	[53]
	BBR	Improve the absorption of cholesterol in the liver and act by improving endothelial dysfunction; Inhibit HMGCoA reductase; ↓ Atherogenic sdLDL	[37,54]
	Polydatin	Cardioprotection by activating myocardial Notch-1/HES1 signaling	[55]
	TSG	Antiapoptotic effect	[56]
	Resveratrol	↓ Balloon-injured arteries	[57]

BBR: BerBeRine; CoQ10: Coenzyme Q10; DHA: DocosaHexaenoic Acid; EGCG: EpiGalloCatechin-3-Gallate; ET-1: EndoThelin-1; GIP: Glucose-dependent Insulinotropic Polypeptide; GLP-1Ras: Glucagon Like Peptide-1 Receptor agonists; HMGCoA reductase: Hydroxy Methyl Glutaryl Coenzyme A reductase; IL: InterLeukin; LDL: Low Density Lipoproteins; LDL-C: Low Density Lipoproteins-Cholesterol LDL-R: Low Density Lipoproteins-Receptor; PCSK9: Proprotein Convertase Subtilisin/Kexin 9; sdLDL: small dance Low Density Lipoproteins; TSG: 9; 2,3,5,4′-TetrahydroxyStilbene-2-O-β-D-Glucoside.

## Data Availability

Not applicable.
